# Posting and transfer: key to fostering trust in government health services

**DOI:** 10.1186/s12960-015-0080-9

**Published:** 2015-10-13

**Authors:** Kabir Sheikh, Lynn Freedman, Abdul Ghaffar, Bruno Marchal, Fadi el-Jardali, Jim McCaffery, Jean-Pierre Olivier de Sardan, Mario dal Poz, Walter Flores, Surekha Garimella, Marta Schaaf

**Affiliations:** Public Health Foundation of India, New Delhi, India; Mailman School of Public Health, Columbia University, New York, USA; Alliance for Health Policy and Systems Research, WHO, Geneva, Switzerland; Institute of Tropical Medicine, Antwerp, Belgium; American University of Beirut, Beirut, Lebanon; TRG, Washington, DC USA; Laboratoire d’études et de recherches sur les dynamiques sociales et le développement local, Niamey, Niger; Social Medicine Institute, University of Rio de Janeiro, Rio de Janeiro, Brazil; Center for the Study of Equity and Governance in Health Systems, Guatemala City, Guatemala; Averting Maternal Death and Disability, 60 Haven Ave., B3, New York, NY 10032 USA

**Keywords:** Health systems governance, Health systems research, Posting and transfer, Qualitative methods

## Abstract

Appropriate deployment or *posting and transfer* (P&T) of health workers – placing the right people in the right positions at the right time – lies at the heart of fostering communities’ faith in government health services and cementing the role of the health system as a core social institution. The authors of this paper have been involved in an ongoing transnational dialogue about P&T practices and determinants. This dialogue seeks to call attention to the importance of P&T as a health system function; to urge donors and policy-makers working in health systems, HRH and public administration governance to consider how to address issues around P&T; and to suggest avenues and approaches to research.

P&T is a vexed and unresolved issue in many low- and middle-income countries that requires, above all, political commitment to improving public sector services and to new thinking and research. It holds promise as a focal point for inter-disciplinary collaboration in research and implementation that can inform other areas in HRH and health systems strengthening. Innovative social science and management theorizing, and iterative, locally driven interventions that focus on establishing transparent professional norms and building the credibility of government administration, including the health services, are likely the way forward.

## Background

Well-functioning government health services are widely recognized to be crucial to achieving universal health coverage (UHC) in low- and middle-income countries (LMICs). Yet, the ambitious plans of many LMICs to achieve UHC are held back by the state of existing government health services. Too often, government health systems fail to provide timely and appropriate services to those most in need.

Though there is enormous variation among and within countries, many health facilities in LMICs, particularly in rural areas, remain unstaffed or understaffed due to the skewed distribution of health workers, inadequate number of trained workers and the drain of qualified health workers away from the public sector into competing employment.

Where health workers are posted, they may not have the appropriate qualifications for the job, or they may report for duty irregularly, feeding the widespread and multifaceted phenomena of worker absenteeism and poor retention.

Absenteeism, poor retention and poor quality of health services are frequently underpinned by health workers’ dissatisfaction with their postings and inadequate support for them to discharge their roles effectively. Women, who make up the bulk of the health workforce, may be disproportionately affected by the social or familial responsibilities that cause absence and challenge retention (see Box 1).

**Box 1:** **A brief description of the definition of posting and transfer**

Posting and transfer (*P&T*) refers to the deployment and transfer of health care workers and administrators. These practices are regulated by the following:	
• formal policies enshrined in public administration structures, such as a public service commission, under the executive branch (for example, the Indian Administrative Service)	
• formal policies particular to the line ministry (MoH) and even to particular cadres. Some decisions, such as intra-district transfer, might be made at decentralized levels, while others, such as recruitment or termination, may be made at the central level	
• practical norms (implicit, tacit norms) [1] relating to political patronage, nepotism, impunity, cronyism, compassion, gendered responsibilities of family caretaking, professional power, multiple accountabilities, etc.	
P&T may be consistent with health worker wishes (for example, bribing a decision-maker to secure an urban post), or they may be inconsistent with these wishes (for example, transferring a poorly performing health worker to a remote rural area). One’s ability to negotiate a preferred outcome likely depends on one’s professional status, with some cadres enjoying more power than others.	
Policies themselves (or the available space to circumvent policy) may be in turn shaped by multiple factors, including lobbying by professional associations/unions and competition among political parties seeking to curry favour with public employee constituencies.	

These health system challenges erode community trust, as patients are faced with empty clinics or poorly treated by frustrated providers. Bypassing and low utilization of facilities are partly caused by users’ distrust of government services and health workers and the resulting expectation of poor quality services [2,3]. Indeed, users frequently complain about corruption and contempt from health personnel [4]. In this vicious circle of poor quality and compromised credibility among communities, government health services often “exist” but fail to attract people in need. Their value in societies is diminished, and vulnerable communities often either go without care or become exposed to poor quality and exploitative practices in the unregulated private health market.

## The role of posting and transfer policy and practice

Appropriate deployment or *posting and transfer* (P&T) of health workers – placing the right people in the right positions at the right time – lies at the heart of fostering communities’ faith in government health services and cementing the role of the health system as a core social institution, capable of providing UHC [5]. P&T is so ubiquitously recognized by health administrators across global LMIC settings as one of the foremost challenges for effective workforce deployment that its relative neglect in the public health literature is resounding. While human resources for health (HRH) as a whole was neglected until recently, P&T remains an under-theorized and under-researched area, including within retention, broader HRH and health systems research. Many causes of current practice and their consequences for the performance of health service delivery in different contexts and for health outcomes are poorly understood. It is a classic example of a tacit ecology of knowledge of crucial importance to public health outcomes that evades the attention of mainstream public health research. In other words, many players on the ground know the real rules of the game, but these rules remain unspoken and are little examined in critical and globally visible research and standard dissemination.

The rules remain unspoken for several reasons. First is the fact that current frameworks of public health and health systems have limited power to interpret and define the challenge, and dominant research methodologies fail to capture its multi-dimensional character. Posting and transfer decisions are usually non-transparent, negotiated outcomes balancing a diversity of interests. Understanding and describing actual P&T practice necessitates going beyond the policy versus practice dichotomy to understand the individual, social, political and structural determinants that shape outcomes [5]. These determinants are not linear contributors but elements of complex adaptive systems. For example, factors such as corruption go well beyond individual decision-making and may stem from emergent characteristics of service delivery and even societies as a whole [6].

In addition, key decision-makers in research agenda setting, including governments and donors, may be reluctant to expose the corrupt and collusive networks that underlie some P&T practices or to surface the extreme divergence between practice and policy. For their part, professional associations or unions may be unwilling to open questions that could ultimately dilute the power that providers have in negotiating P&T.

The authors of this paper have been involved in an ongoing transnational dialogue about P&T practices and determinants. This dialogue seeks to call attention to the importance of P&T as a health system function; to urge donors and policy-makers working in health systems, HRH and public administration governance to consider whether and how to address P&T; and to suggest avenues and approaches to research. Beginning with a regional multi-stakeholder discussion in New Delhi in 2013, we explored whether it was feasible and relevant to do empirical research on P&T. Participants discussed the particularities of British colonialism, post-independence state building in South Asia, regulation and the health system, patronage and political competition, how well the dynamics of P&T are captured in current discussions of retention and public management and variations in P&T determinants and practice within and among countries. Wishing to further explore commonalities and differences as well as global opportunities to advance P&T as an issue, the group convened a global discussion at the Rockefeller Foundation’s Bellagio Center in 2014.

The Bellagio group fleshed out the local particularities and the global commonalities of P&T. Indeed, typifying complex adaptive systems, P&T is inextricably linked to a range of factors specific to the setting. Prevailing official and practical norms of public administration and management, labour market dynamics [7], human resource management systems and accountability mechanisms within health systems all impinge on how P&T policies are framed, interpreted and implemented. The propagation of many so-called “best practices” in public sector administration and human resource management has seemingly failed to affect the prevailing P&T practice. Unfortunately, inappropriate P&T is all too common and is linked to an informal trade of “lucrative” postings and frequent political, clientelistic and nepotistic interventions in the P&T process. For example, in Niger, around 90% of the midwives are posted in Niamey and the big towns, as they have secured protection from high-level civil servants [8,9]. Similarly, in Guatemala, district parliamentarians have functional control over the hiring of public service personnel, despite laws recognizing this as illegal interference with the executive branch of government [10]. The ability to obtain a preferred posting likely varies significantly by cadre.

Conversations grew and deepened at the Third Global Symposium on Health Systems Research, held in Cape Town, South Africa, in 2014. There, we agreed that realizing the promise of UHC and fostering trust in health services will necessitate examination of neglected and persistent challenges related to P&T and linking these discussions to debates and forums focusing on HRH, retention and other related areas.

These deliberations highlighted that the drivers of prevailing P&T practice, as well as the means to strengthen P&T, are situated not only in technical and policy solutions but in transforming the norms, values, practices and relationships that underpin health services performance, health professional associations/unions and the public administration more broadly. These intangible elements – the “software” [11] of the public sector – are critical to understanding and reforming P&T.

Participatory and qualitative research methods grounded in a range of different social science disciplines hold promise in being able to explicate and uncover the complex social, political and organizational dynamics at play in P&T. While some of these approaches have been effectively used in research on retention, human resource management and other fields, our multi-disciplinary group of researchers and practitioners believes that the frontiers of these fields can be expanded to better accommodate ethnographic insights, non-linearity and emergence and the relationship between public sector management, professional power and political competition and control. There are also a host of micro-contextual issues that we know little about, such as how P&T dynamics differ among different cadres, how the scope of clinical practice shapes health worker ability and desire to negotiate postings and whether or not punitive transfers can be effective as an accountability or human resource management tool.

The global health community needs to awaken to the primacy of this problem and the need to nurture the foundation of values that will sustain the core role and function of government health services as social institutions, with the trust of the communities that they serve. While P&T is only one factor in shaping trust and UHC, its longstanding neglect and its embedded nature mean that it is an important factor. Moreover, P&T can be understood to be a tracer for broader issues of germane significance that are shaped by health systems software.

## Conclusion

P&T requires, above all, political commitment to improving public sector services and to new thinking and research. It holds promise as a focal point for inter-disciplinary collaboration in research and implementation that can inform other areas in HRH and health systems strengthening. Innovative social science and management theorizing, and iterative, locally driven interventions that focus on establishing transparent professional norms or more efficient practical norms and building the credibility of public administration, including health services, are likely the way forward.

## Abbreviations

UHC: Universal health coverage
LMICs: Low- and middle-income countries
P&T: Posting and transfer
HRH: Human resources for health
